# Job strain — Attributable depression in a sample of working Australians: Assessing the contribution to health inequalities

**DOI:** 10.1186/1471-2458-8-181

**Published:** 2008-05-27

**Authors:** Anthony D LaMontagne, Tessa Keegel, Deborah Vallance, Aleck Ostry, Rory Wolfe

**Affiliations:** 1 McCaughey Centre: VicHealth Centre for the Promotion of Mental Health & Social Wellbeing School of Population Health, University of Melbourne Melbourne, VIC 3010, Australia; 2 Faculty of Social Sciences University of Victoria, Victoria, BC, Canada; 3 Department of Epidemiology & Preventive Medicine Faculty of Medicine, Nursing and Health Sciences Monash University at Alfred Hospital Melbourne, VIC 3004, Australia

## Abstract

**Background:**

The broad aim of this study was to assess the contribution of job strain to mental health inequalities by (a) estimating the proportion of depression attributable to job strain (low control and high demand jobs), (b) assessing variation in attributable risk by occupational skill level, and (c) comparing numbers of job strain–attributable depression cases to numbers of compensated 'mental stress' claims.

**Methods:**

Standard population attributable risk (PAR) methods were used to estimate the proportion of depression attributable to job strain. An adjusted Odds Ratio (OR) of 1.82 for job strain in relation to depression was obtained from a recently published meta-analysis and combined with exposure prevalence data from the Australian state of Victoria. Job strain exposure prevalence was determined from a 2003 population-based telephone survey of working Victorians (n = 1101, 66% response rate) using validated measures of job control (9 items, Cronbach's alpha = 0.80) and psychological demands (3 items, Cronbach's alpha = 0.66). Estimates of absolute numbers of prevalent cases of depression and successful stress-related workers' compensation claims were obtained from publicly available Australian government sources.

**Results:**

Overall job strain-population attributable risk (PAR) for depression was 13.2% for males [95% CI 1.1, 28.1] and 17.2% [95% CI 1.5, 34.9] for females. There was a clear gradient of increasing PAR with decreasing occupational skill level. Estimation of job strain–attributable cases (21,437) versus "mental stress" compensation claims (696) suggest that claims statistics underestimate job strain–attributable depression by roughly 30-fold.

**Conclusion:**

Job strain and associated depression risks represent a substantial, preventable, and inequitably distributed public health problem. The social patterning of job strain-attributable depression parallels the social patterning of mental illness, suggesting that job strain is an important contributor to mental health inequalities. The numbers of compensated 'mental stress' claims compared to job strain-attributable depression cases suggest that there is substantial under-recognition and under-compensation of job strain-attributable depression. Primary, secondary, and tertiary intervention efforts should be substantially expanded, with intervention priorities based on hazard and associated health outcome data as an essential complement to claims statistics.

## Background

Job stress is a risk factor for a range of adverse effects on health, including major contributors to the overall burden of disease in developed countries, such as cardiovascular disease (CVD) and depression [1-4]. Previous estimates of the proportion of various diseases attributable to occupational exposures have been made by combining population-based exposure prevalence data with estimates of exposure-related increases in specific disease risks taken from epidemiologic studies, yielding exposure-specific 'population attributable risks' (PAR) [5]. PAR is the proportion of disease cases attributable to the exposure in question, or the fraction of disease cases that would *not *have been observed if the exposure was non-existent [6].

Previous PAR estimates for job stress have utilized Karasek and Theorell's demand-control measures of job stress [7] in relation to cardiovascular disease (CVD) mortality [5,8,9]. We found only one estimate of job stress-related PAR for depression, from a Finnish study [9]. Using the prevalence estimates for job strain of 19% for men and 23% for women, this study estimated that 14.6% of deaths related to 'depressive episodes' among men and 9.8% among women were attributable to job strain [9]. While a focus on mortality in work-related PAR estimation may be suitable for CVD and cancer, morbidity estimates would be more useful for mental illness. A recent WHO global quantitative risk assessment initiative generated integrated morbidity and mortality disease burden estimates (disability-adjusted life years) for selected occupational risk factors and associated disease outcomes, but these did not include psychosocial working conditions or mental health outcomes [10].

In the present study, we seek to extend previous work by estimating PAR for depression, and examining variation in job strain exposure and associated attributable disease risk across occupational skill levels as a measure of social class [11]. We have focused on depression because it is a growing contributor to the general burden of disease, and is the most prevalent form of mental illness in developed countries [12]. Examination of exposure and related disease patterns by occupational level contributes to the growing awareness and investigation of psychosocial and other working conditions as important contributors to health inequalities [1,11,13].

## Methods

Effect size estimates for job strain in relation to depression were extracted from the published literature, and combined with exposure prevalence data from the Victorian Job Stress Survey to estimate the proportions of depression attributable to job strain. These proportions were then applied to prevalent cases of depression to yield numbers of job strain-attributable cases of depression. Further, to determine the extent to which regularly collected claims data captures work-related depression or other mental health conditions, we compared our prevalence estimates with the number of compensated "mental stress"-related workers compensation claims. Data sources for each are described below.

### Magnitude of increased risks of depression from job strain

A recent meta-analysis assessed relationships between common mental disorders and various demand-control model measures (job control, job demands, job strain, and social support at work) [4]. For job strain, two studies met inclusion criteria and yielded a combined OR of 1.815 (95% CI 1.062, 3.101). One was a large prospective population-based Canadian study in which job strain showed different association with depression for men (adjusted OR 3.30 [1.29, 8.44] versus women (aOR 2.10 [1.10, 4.00]) [14]. In contrast, the second study, a longitudinal Finnish cohort of 4815 hospital personnel, found no association between job strain and depression, although there were significant associations between organisational justice and depression [15]. Both studies adjusted for age and income, with each adjusting further for unique sets of covariates. Though some of the numerous meta-analyses reported single effect size estimates for a variety of common mental disorders as one outcome (e.g., anxiety, depression, psychological distress) [4], the two studies relied upon in the job strain analysis both used incident cases of depression as an outcome–12-month depression as determined by CIDI in one study [14], and new doctor-diagnosed clinical depression in the other [15]. Both studies excluded baseline cases of depression from analysis. Thus, the job strain effect size reported in this meta-analysis is most suitable for PAR estimation in relation to incident depression as an outcome.

### The Victorian Job Stress Survey

The Victorian Job Stress Survey (VJSS) was conducted by telephone from a random sample of White Pages listings in the southeastern state of Victoria, Australia. The protocol for this study was reviewed and approved by the University of Melbourne Human Research Ethics Committee (HREC #030398). Quota sampling was conducted to reflect Australian Bureau of Statistics (ABS) census proportions of upper white-collar, lower white-collar, and blue-collar groups (41%, 30%, and 29%, respectively), as well as urban Melbourne (72%) versus rural/regional Victoria (28%). Inclusion criteria were being aged 18 years or older, and working at the time of the survey for profit or pay (including self-employed). Interviews were completed in November 2003 with a 66% response rate from in-frame households (i.e., had one or more residents aged 18 or over and working) to yield a representative sample of 1,101 working Victorians (526 men and 575 women), as described in detail previously [16].

#### Job stress measures

Karasek & Theorell's demand and control measures [7] were assessed as the sum of 3 items for psychological demand (Cronbach's alpha = 0.66), and the combination of two equally weighted scales of 6 and 3 items measuring skill discretion and decision authority respectively for job control (Cronbach's alpha = 0.80), as previously described [16]. Demand and control measures were dichomtoised at the median, and combined to create categories of low strain (low demand and high control), active jobs (high demand and high control), passive jobs (low demand and low control), and job strain (high demand and low control). In subjects with missing data, scores were recalculated using the lower and the higher theoretical score for each missing item and dimensions dichotomised according to their median. If the classification of participants was the same for any possible value of the missing item, participants were considered as having non-missing answers for the dimension of interest (38/88 participants with missing data). If the classification differed according to the replaced value, participants were considered as having a missing answer for the dimension [17]. Non-missing job strain measures were thus calculable for a total of 501 men and 550 women.

#### Covariates

Covariate data were collected for a range of demographics as described previously including occupational skill level, age, and highest level of education completed. Occupational skill levels were collapsed from nine into five Australian Bureau of Statistics (ABS) skill levels, from one (highest skill) to five (lowest skill) [16]: managers, administrators and professionals (level 1), associate professionals (level 2), tradespersons and related workers, and advanced clerical and service workers (level 3), intermediate clerical, sales, and service workers, and intermediate production and transport workers (level 4), elementary clerical, sales and service workers, and labourers and related workers (level 5).

### Prevalence of depression among working Victorians

Twelve-month depression prevalence estimates among employed Australians were obtained from the 1997 Australian Bureau of Statistics (ABS) National survey of Mental Health and Wellbeing [18]. These were the most recent estimates available, determined as DSM-IV depression using a modified Composite International Diagnostic Interview (CIDI) (3.89% among employed men, and 8.41% among employed women, provided by Dr. Kristy Sanderson). These national prevalence estimates were applied to the absolute number of employed Victorian men and women obtained from published 2001 ABS Labour Force statistics [19]. This estimate of the number of 12-month depression cases among employed Victorians was the closest available to the 2003 survey from which job strain prevalence was estimated.

### Stress-related workers compensation claims among working Victorians

The former National Occupational Health and Safety Commission (NOHSC), now the Australian Safety & Compensation Council (ASCC), previously compiled and made publicly accessible a national workers' compensation (WC) statistics database [20]. Numbers of cases were derived from WC claims received from insurance companies, self-insurers, and all levels of government. This database was queried for Victorian job stress claims for the same as year as the VJSS (2003), with stress-relatedness identified by a "mechanism of injury or disease classification" of "mental stress" [21].

### Statistical analysis

Differences in job strain prevalence due to gender and occupational skill level were summarised as Odds Ratios (OR) using multiple logistic regression (outcome = presence of job strain), with occupational skill level treated as an ordinal variable. Population attributable risks (PAR) were calculated according to the formula PAR = (p * [OR - 1]/1 + p * [OR - 1]) * 100, where p = prevalence of exposure and OR = associated outcome effect size [22]. Data analysis was performed using STATA 9.0 (Stata Corporation, College Station, TX).

## Results

Job strain prevalence was significantly higher among women than men (25.5% versus 18.6%), including after adjustment for occupational skill level (Table 1, univariate and multivariate columns). Job strain prevalence increased approximately 20% with each unit of decreasing skill level (Table 1, univariate and multivariate columns). There was a statistically significant increase in job strain prevalence with decreasing occupational skill level in the population as whole, as well as in each gender separately (Table 1, third and fourth columns). While in our sample this relationship was observed to be stronger in men we cannot rule out the possibility of equal relationships in males and females generally (test of gender by skill level interaction, p = 0.49). PAR estimates were calculated separately for these important demographic determinants of job strain prevalence, i.e., sex and occupational skill level, to enable exploration of the potential contribution of job strain to health inequalities.

**Table 1:** Population Patterns of Job Strain by Gender and Occupational Skill Level: Logistic Regression Results as Odds Ratios with 95% Confidence Intervals (OR [95% CI])

	**Univariate OR [95% CI] N = 1051**	Multivariate* OR [95% CI] N = 1051	**Men Only OR [95% CI] N = 501**	**Women Only OR [95% CI] N = 550**
**Gender (male = reference)**	1.50 [1.11, 2.01]	1.53 [1.13, 2.06]	--	--
**Occupational skill level (1–5, highest to lowest)**	1.20 [1.08, 1.32] p < 0.001 (test for trend)	1.20 [1.09, 1.33] p = 0.000 (test for trend)	1.26 [1.07, 1.49] p = 0.006 (test for trend)	1.17 [1.03, 1.33] p = 0.014 (test for trend)

* Gender and skill level OR adjusted for eachother.

The point estimate for job strain-attributable depression among working men was 13.2% (Table 2), with upper limit of approximately 1 in 4 cases (95% CI 1.1, 28.1). The overall estimate in women was higher (Table 2), 17.2%, with an upper limit of approximately 1 in 3 cases attributable to job strain (95% CI 1.5, 34.9).

**Table 2:** Estimated Cases of Depression Attributable to Job Strain in Victorian Working Population

	**Victorian employed persons (ABS, 2001)**	**Prevalence of 12-month depression in Australian employed persons (ABS, 1997)**	**Cases of 12-month depression, employed persons**	**Percent of 12-month depression cases attributable to job strain**	**Depression cases attributable to job strain**
**Males, age 15–64**	1,302,500	3.89%	50,667	13.2%	6,688
**Females, age 15–64**	1,019,600	8.41%	85,748	17.2%	14,749

**Figure 1:**
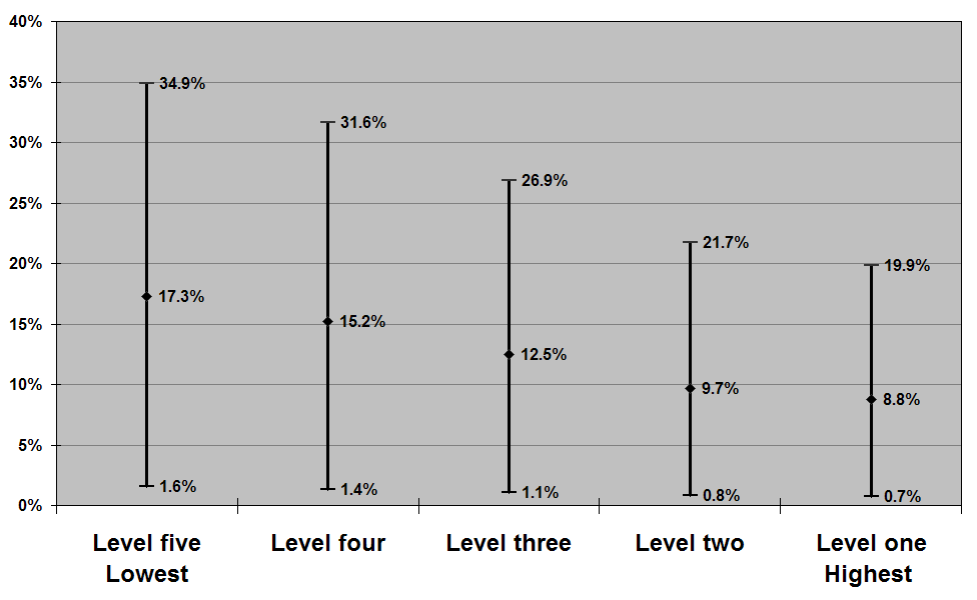
Population Attributable Risk Estimates for Job Strain in Relation to Depression among Working Victorians, by Occupational Skill level: Males, n = 501 (point estimate & 95% confidence interval).

**Figure 2:**
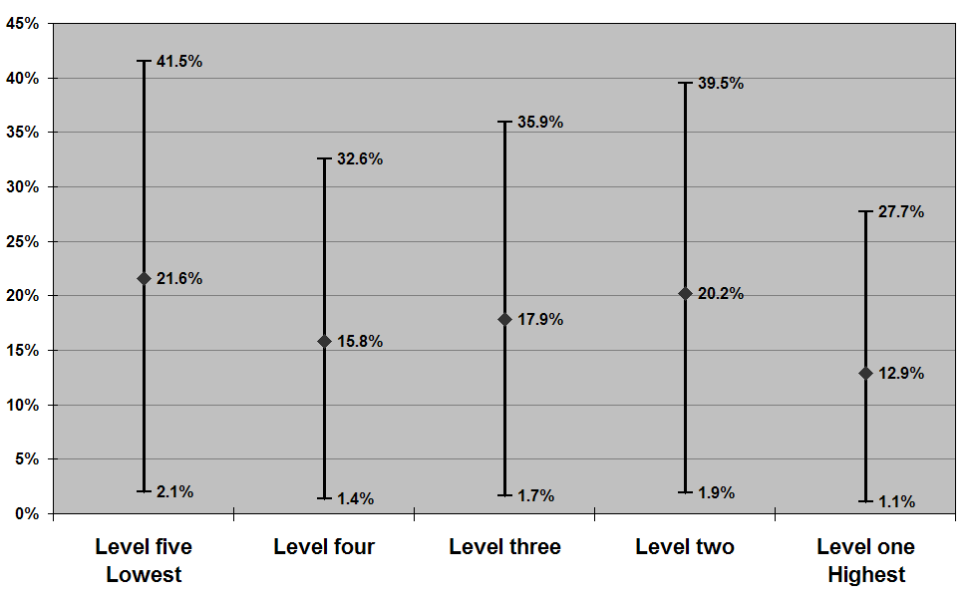
Population Attributable Risk Estimates for Job Strain in Relation to Depression among Working Victorians, by Occupational Skill level: Females, n = 550 (point estimate & 95% confidence interval).

Job strain-attributable depression estimates by occupational skill level are presented in Figures 1 and 2 (see additional files 1 and 2). Among men, there was a statistically significant, stepwise linear trend (Table 1) in job strain exposure from the highest skill level to the lowest (11.8, 13.2, 17.5, 22.0, and 25.6% respectively), and thus a corresponding gradient in PAR for depression (Figure 1, see additional file 1). Point estimates span a two-fold range of roughly 17 to 9 percent from the lowest to highest skill-level working men. While job strain prevalence showed a significant linear trend among women (Table 1), there was not a clear stepwise gradient of exposure (18.2, 31.1, 26.7, 23.0 and 33.8% from highest to lowest skill levels), and thus a less clear gradient in PAR estimates (Figure 2, see additional file 2). Nevertheless, as observed in men, the point estimates for women approximate a doubling of attributable risk, from 22 to 13 percent, going from the lowest skill level to the highest.

### Prevalence of depression among working Victorians

Table 2 presents estimates of 12-month depression prevalence among working Victorians (third column, calculated directly from columns 1 and 2). Applying the job strain-attributable proportions from above (fourth column) to these figures yields estimates on the order of 6600 job strain-attributable cases of depression among working age Victorian males, and more than twice that number among working Victorian females (far right column).

### Stress-related claims among working Victorians

A total of 1723 "mental stress" WC claims were reported for 2003: 788 for males and 935 for females. The nature of these claims is further elucidated by a government analysis of "mental stress" claims nationally from 2004–2005 [21]. This analysis identified the most common sub-category of "mental stress" claims as involving "work pressure" (3305 claims representing 41% of total), defined as disorders arising from work responsibilities and workloads, workplace interpersonal conflicts and performance and promotion issues [21]. This was followed by "harassment" (1730 claims, 22%), and violence (1300 claims, 16%). Accordingly, we would expect approximately 41% of "mental stress" claims to be related to chronic stressors such as job strain, yielding estimates of 323 (0.41 * 788) compensated claims for males, and 383 (0.41 * 935) for females. These estimates suggest that claims statistics underestimate job strain-attributable depression by roughly 30-fold (696 compensated versus 21,437 estimated).

## Discussion

Estimated proportions of depression attributable to job strain among working Victorians indicate that job stress is a substantial public health problem. Findings also show that job strain and associated depression risks are inequitably distributed, with workers in lower skill level jobs most likely to be adversely affected, particularly among males. Both in Victoria and in industrialised democracies internationally, poor mental health is disproportionately prevalent among those in lower status occupations, and with lower educational attainment and lower incomes [23-26]. Our findings suggest that job strain may be an important contributor to these mental health inequities.

Our job strain–attributable depression estimates are in the same range to those previously published for job strain in relation to cardiovascular disease (7–16% among men for job strain assessed at a single point [8]) and coronary heart disease (16% in men and 19% in women [9]). Taken together, job strain-attributable risks for these two major chronic diseases represent a substantial preventable disease burden. With respect to the burden of occupational disease more broadly, this study complements and extends previously published Australian mortality estimates for occupational exposure to hazardous substances [27] and morbidity estimates for occupational cancers [28].

The PAR estimates presented are conservative in several ways. The analyses are based on a conservative effect size estimate that is lower than those published for most individual studies. Some cross-sectional studies have found strong associations between job strain and prevalent cases of depression, such as a US study that presented adjusted ORs for job strain and major depressive episode (OR = 7.0), depressive episode (OR = 4.1), and dysphoria (OR = 2.9) among women, with no significant associations among men [29]. Longitudinal studies, by contrast, tend to find smaller effect sizes for incident cases. In a 4-year longitudinal study of depression outcomes in Swedish workers that also accounted for the role of non-occupational factors such as coping ability and stressful life events, job strain remained significantly associated with sub-clinical depression (RR = 2.8) for women, but not for men [30]. The French longitudinal GAZEL study found that the demand/control model measures of high psychological demands (OR = 1.77 men, 1.37 women), low job control (OR = 1.38 men, 1.41 women), and low social support (OR = 1.58 men, 1.29 women) predicted subsequent depressive symptoms at 1-year follow-up [31]. All effects were statistically significant and were unchanged after adjustment for potential confounders. Similar results were found on 3-year follow-up [32]. These investigators did not assess job strain as a predictor variable, but found similar effects of demand/control variables for men and women. Another French (cross-sectional) study of a different population found that job strain was a significant risk factor for depressive symptoms for men (OR = 1.94), but not for women [33]. This finding was largely attributed to job control, which was a significant risk factor for depressive symptoms (OR = 2.84) as well as psychiatric disorders (OR = 2.02) in men [33]. The literature includes a limited number of Australian studies. In a sample of 40–44 year old workers (n = 2249 from low, middle and high status jobs), a statistically significant cross-sectional association was observed between job strain and depression (OR= 2.46) for men and women [34]. Another analysis of a subset of 1,188 employed professionals from the same sample found an OR of 2.54 for depression, again for men and women combined [35]. These cross-sectional Australian estimates are similar to those obtained internationally from longitudinal studies. Finally, we would note that for the purposes of our Victorian working population PAR estimates, effect size estimates would most appropriately come from population-based studies and not studies of specific occupational groups with more restricted generalisability. Of the two studies on which the meta-analysis was based, only one was population-based: the Canadian study in which job strain showed different associations with depression for men (aOR 3.30) versus women (aOR 2.10) (further details in Methods section above). The second was from a longitudinal Finnish cohort of hospital workers, which found no association between job strain and depression. The less relevant study (for the purposes of the analyses presented in this paper) brought the meta-analysis effect size estimate down to 1.8. The net effect of using the meta-analysis effect size estimate in our calculation is that we are more likely to be under-estimating than over-estimating job strain–attributable depression in the Victorian working population.

The PAR estimates presented are also conservative in that the steepness of the gradient observed may be underestimated. It is postulated that job stress has a greater impact on mental health among people in lower socioeconomic positions [11]; as has been empirically demonstrated with respect to hypertension and CVD [11,36]. In addition, to produce a comprehensive estimate of the effects of job strain on mental health, other associated mental health outcomes would need to be included, such as anxiety, work-related suicide, and behavioural disorders (for example, alcoholism and nicotine addiction) [4,37,38]. Further, job strain represents only one of several work-related psychosocial hazards. Others that have been linked to depression include effort-reward imbalance, injustice at work, job insecurity, and bullying [4,39,40]. All such hazards would need to be included to estimate the full effect of psychosocial work hazards on depression in particular, and on mental health disorders in general. Thus, we would argue that the impact of *all *psychosocial working conditions on depression would be higher than the estimates we have presented, and corresponding estimates for *all *affected mental health outcomes would be higher still.

We also acknowledge certain limitations of this study. Although the survey was designed to be representative of the working population, the sample was taken from telephone listings. This may disproportionately exclude those workers who are in less secure employment, in lower status groups, shift workers and those working longer hours. This bias would lead to underestimation of differentials in exposure and disease distributions across occupational levels. Further, job strain has been studied in relation to various measures of depression, including the CES-D scale [32,33], Goldberg's 9-item scale [34], major depressive episode as determined by CIDI method [14], and doctor-diagnosed clinical depression [15]. The effect size estimate and the ABS depression prevalence estimates used in this analysis, however, were both based on 12-month depression and included the use of CIDI measurement method. In addition, there are underlying assumptions for the PAR methods used [6]. We combined local exposure prevalence data with an effect size estimate from international studies. This approach assumes that disease aetiology and co-exposures internationally will be similar to the Australian context. This assumption is reasonable because: 1) studies relied upon for effect size estimation came from developed countries, 2) adjusted effect sizes across the various developed countries studied were similar, 3) adjusted (cross-sectional) Australian estimates are consistent with and higher than the effect size used.

We also acknowledge the need for further study of job stress in general and job strain in particular in relation to mental health outcomes. Though the majority of studies of job strain or its component scales of job control and job demands show positive associations, studies of job strain and depression show inconsistent effect sizes, with some studies finding a null association. Substantial differences in the effect size estimates for the contributing studies to the meta-analysis used in the PAR calculations led to its wide confidence intervals (1.815 [95% CI 1.062, 3.101]), and in turn to wide confidence intervals in our PAR estimates. But we also argue from a precautionary principle perspective that the evidence presented is adequate to justify expanded policy and practice responses, particularly given the scale of the problem and the availability of intervention strategies to address it [41]. Several reviews of the intervention evaluation literature have demonstrated that job stress can be effectively addressed by comprehensive or systems approaches–that is, combining individual- and organisation-directed, or work- and worker-directed intervention strategies [42-46].

Finally, comparison of job strain-attributable depression estimates to compensated "mental stress" claims shows that the scale of the problem is grossly underestimated by WC statistics. There are a number of contributing factors for this discrepancy. WC statistics are based on accepted claims. Information regarding the numbers of claims submitted is unavailable. However, given the adversarial nature of the WC system, it is likely that many workers with stress-related illnesses, including depression, have their claims rejected, or may be deterred from filing a claim. Further, when a worker presents to a medical practitioner with depression, the practitioner may or may not identify an underlying occupational causation or contribution. Even if job stress is medically recognized as a contributory factor, there is a documented reluctance amongst Australian general practitioners to initiate WC claims for patients presenting with job stress-related conditions [47,48]. Further reinforcing this point, a study of Australian human services workers found that psychological claims were four times more likely to be rejected than other kinds of claims, and that the investigation process was reported by claimants to be particularly stressful [49].

## Conclusion

This discrepancy between epidemiologic versus insurance-based estimates of job strain–attributable depression demonstrates an urgent need for population-based exposure and health data to complement claims statistics as a basis for guiding policy and practice strategies and priorities in this area. Further, the social patterning of job strain-attributable depression indicates that concerted action by occupational health & safety regulatory agencies and other workplace stakeholders, particularly in lower-skilled work contexts, has the potential to reduce mental health inequities on a population level. Applying a health inequalities intervention framework consistent with the systems approach described above, Whitehead recently described how stressful psychosocial working conditions can be addressed by a combination of strengthening individuals, strengthening communities, improving living and working conditions, and promoting healthy macro-policies [50]. There is an urgent need and a significant opportunity to better link work and health concerns and intervention strategies with the more broadly embraced efforts to understand and address health disparities.

Given the availability of feasible and effective intervention strategies, our findings add to the growing evidence base in support of expanded public health policy and practice interventions to address the adverse effects of job stress. A substantial and inequitable disease burden could be addressed by applying a systems approach to job stress in Victoria and elsewhere. In addition to being a concern for workers, unions, employers, occupational health and safety, and workers' compensation systems, job stress should be a concern for physical and mental health promotion agencies, government public health authorities (and health departments), medical practitioners, community advocacy groups, and others. An optimal public health response to job stress would encompass participation by the full range of stakeholders.

## Competing interests

The authors declare that they have no competing interests.

## Authors' contributions

ADLaM conceived of the study, directed analyses, and led the writing of the paper. TK and DV assisted with the conceptualisation, analysis, and writing. AO and RW assisted with conceptualisation and analyses. All authors contributed to the interpretation of results, refinement of analyses, and review of successive drafts of the manuscript.

## Pre-publication history

The pre-publication history for this paper can be accessed here:


